# Hyaluronic acid-conjugated liposome nanoparticles for targeted delivery to CD44 overexpressing glioblastoma cells

**DOI:** 10.18632/oncotarget.8926

**Published:** 2016-04-22

**Authors:** Stephen L. Hayward, Christina L. Wilson, Srivatsan Kidambi

**Affiliations:** ^1^ Department of Chemical and Biomolecular Engineering, University of Nebraska-Lincoln, Lincoln, NE, 68588, USA; ^2^ Nebraska Center for Materials and Nanoscience, Lincoln, University of Nebraska-Lincoln, Lincoln, NE, 68588, USA; ^3^ Nebraska Center for the Prevention of Obesity Diseases, University of Nebraska-Lincoln, NE, Lincoln, 68583, USA; ^4^ Mary and Dick Holland Regenerative Medicine Program, University of Nebraska Medical Center, Omaha, NE, 68198, USA; ^5^ Fred and Pamela Buffett Cancer Center, University of Nebraska Medical Center, Omaha NE, 68198, USA

**Keywords:** glioblastoma, nanomedicine, CD44 targeting, lipid nanoparticles, hyaluronic acid

## Abstract

Glioblastoma Multiforme (GBM) is a highly prevalent and deadly brain malignancy characterized by poor prognosis and restricted disease management potential. Despite the success of nanocarrier systems to improve drug/gene therapy for cancer, active targeting specificity remains a major hurdle for GBM. Additionally, since the brain is a multi-cell type organ, there is a critical need to develop an approach to distinguish between GBM cells and healthy brain cells for safe and successful treatment. In this report, we have incorporated hyaluronic acid (HA) as an active targeting ligand for GBM. To do so, we employed HA conjugated liposomes (HALNPs) to study the uptake pathway in key cells in the brain including primary astrocytes, microglia, and human GBM cells. We observed that the HALNPs specifically target GBM cells over other brain cells due to higher expression of CD44 in tumor cells. Furthermore, CD44 driven HALNP uptake into GBM cells resulted in lysosomal evasion and increased efficacy of Doxorubicin, a model anti-neoplastic agent, while the astrocytes and microglia cells exhibited extensive HALNP-lysosome co-localization and decreased antineoplastic potency. In summary, novel CD44 targeted lipid based nanocarriers appear to be proficient in mediating site-specific delivery of drugs via CD44 receptors in GBM cells, with an improved therapeutic margin and safety.

## INTRODUCTION

Glioblastoma Multiforme (GBM) is the most aggressive, lethal, and prevalent brain malignancy with over 10,000 new cases diagnosed in the United States each year [1, 2] GBM is specifically a grade IV astrocytoma histologically defined by abnormal cellularity, mitotic activity, vascular proliferation, and necrosis leading to a highly mobile and invasive phenotype capable of infiltrating surrounding brain tissue [3]. Consequently, GBM is characterized by poor prognosis, restricted disease management potential, and a less than 1 year median survival rate [4]. Current widespread clinical treatment options for GBM include radiation therapy, chemotherapy with antineoplastic agents, and maximal tumor resection [5]. However, these treatment measures instigate systemic toxic effects to healthy tissue, are limited in potency by intrinsic resistance pathways, require regular invasive dose regimens, and overall do not provide improved long-term quality of life for the patient. Therefore there is a critical need to develop a novel approach that can overcome current limitations and alleviate the burden of GBM.

Brain tumors are categorized according to the glial type they are most histologically similar, location of the tumor, and overall phenotypical behavior [6]. Brain tumor microenvironment also consists of other cells including glial cells (astrocytes and microglia) that are a class of non-neuronal brain cells. Astrocytomas including GBM are most similar to astrocytes, and therefore it is crucial to ensure that any targeting approach developed for GBM treatment must be able to 1) distinguish between GBM cancer cells and healthy astrocytes, and 2) evade rapid phagocytosis by microglial cells. A promising method for active targeting to cancer cells is the exploitation of the differential expression of CD44. CD44 is a cell membrane-bound surface receptor that mediates cell-cell and cell-extracellular matrix (ECM) communication [7–11], and has been found to be increased in numerous cancer types including breast [12], lung [13], colorectal [14] tumors compared to basal expression in equivalent healthy tissue [15]. Hyaluronic acid (HA), a main component of the ECM, is a natural ligand to CD44 has been used as a targeting moiety for CD44 overexpressing cancers, facilitating preferential uptake and potent therapeutic efficacy [16–22]. Recently, CD44 has also been found to be increased in glioma cells compared to healthy astrocytes [23, 24], and has been implicated to directly impact glioma invasion [24, 25]. Although these findings are extremely exciting, only a few studies involving an HA decorated nanocarrier have been examined as a potential CD44 targeted GBM therapy [26, 27]. Furthermore, no studies to date have performed an in-depth analysis probing the true merit of HA as a natural ligand to preferentially bind and internalize into GBM cells over healthy glial cells.

Recently, liposomes and lipid-based nanocarriers have demonstrated robust efficacy in drug and gene therapy comprising precise coordinates of the body such as the brain [28–31]. The successful application of liposome nanocarriers has been catalyzed by targeted delivery and subsequent preferential intracellular uptake via either passive (diffusion driven accumulation and local cell uptake) or active (explicit cell receptor driven uptake) mechanisms [32–37]. Although passive approaches have been employed extensively via implementation of the enhanced permeability and retention (EPR) effect, the advent of biomarker identification coupled to pathological categorization has greatly improved the efficacy, selectivity, and overall safety of liposome therapeutic delivery. From this approach, liposomes can be surface decorated with receptor recognition ligands including antibodies, aptamers, peptides, and integrins to facilitate targeting of specific biomarkers that are identified in disease states. For example, EGFR [38], Folate Receptor [39, 40], and HER2 [41] directed nanotherapies have significantly improved global cancer treatment via cell specific endocytosis of therapeutic cargo. In regards to GBM therapy, a wide array of active recognition ligands have been utilized to date including the peptide sequences IL13 [42, 43], CGKRK [44], Pep1 [45], activatable low molecular weight protamine [46], and chlorotoxin [47], antibodies such as EGFRvIII [48], low density lipoprotein receptor related proteins [49, 50], and the GMT8 aptamer [51]. These targeting moieties have exploited GBM cell specific membrane signatures with varying levels of success to deliver numerous drug types including the chemotherapeutics doxorubicin [43] and paclitaxel [49], antisense oligonucleotides/silencing RNAs [4, 47], peptide based therapeutics such as KLAKLAK [44], inhibitors [52, 53], and other therapeutic cargo types [54–56] for GBM management. While encouraging, there is limited knowledge on how the different cell types in the brain, specifically glial cells, influence the uptake selectivity of active GBM targeted therapy.

In this study we investigated the potential of implementing HA as a surface bound targeting moiety for liposome GBM therapy. To do so, we employed our previously optimized high molecular weight HA conjugated liposomes (HALNPs) to study the uptake pathway in key cells in the brain including primary astrocytes, microglia (MG), and human GBM cells [22, 57]. Specifically, three GBM cells lines were utilized: invasive and non-tumorigenic (A172 cells), non-invasive and slightly tumorigenic (U251), and invasive and highly tumorigenic (U87MG). From this approach, we determined the rate and overall extent of HA coated liposome intracellular delivery to astrocytes, microglial, and GBM cells, as well as probed the capacity of each cell type to implement CD44 stimulated uptake and the resultant effect on nanoparticle endolysosomal fate. A potency assay employing doxorubicin in both the free form and encapsulated inside HA coated liposomes was also performed to validate the robust influence of targeted therapy on the selectivity and efficacy of GBM treatment. We believe this information is significant in the development of novel GBM nano-therapies, and will catalyze the usage of CD44 targeting for the treatment of GBM.

## RESULTS AND DISCUSSION

### Design and characterization of hyaluronic acid coated liposomes (HALNPs)

**Figure 1:**
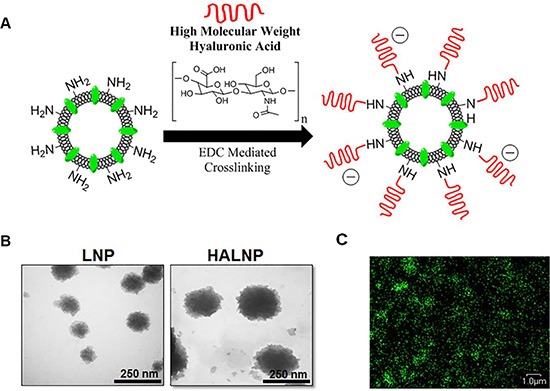
Hyaluronic acid (HA) decorated lipid nanocarrier fabrication overview and characterization (**A**) Lipid nanoparticles (LNPs) were surface functionalized with HA (HALNPs) via EDC facilitated amide bond formations. The LNPs were characterized via (**B**) transmission electron microscopy (TEM) pre and post HA surface crosslinking to confirm significant surface decoration, and (**C**) by high magnification confocal microscopy to illustrate the fluorescent nature of the HALNPs due to the lipid bilayer incorporated fluorescent tracker (495 ex.; 520 em.). The TEM scale bars at 250 nm and the scale bar for the confocal microscopy is 1 micron.

To investigate the potential of using hyaluronic acid (HA) as a targeting ligand for GBM therapy, we first engineered an HA conjugated nanocarrier system following our previously optimized protocol [22, 57]. The liposome nanoparticles (LNPs) were made from the biocompatible constituents PC, DPPE, and CHOL in a 3:1:1 molar ratio and doped with 0.15 mass % fluorescent cholesterol as a tracker. The LNPs were surface decorated with high molecular weight HA (HALNPs) via an EDC facilitated amide bond formation between the carboxyl group of the HA and the primary amine of the DPPE lipid head group as seen in Figure 1A. Initial validation of significant surface functionalization was determined by size and charge characterization (Table 1). Bare LNPs were 95.0 ± 0.7 nm in hydrodynamic diameter with a polydispersity index (PI) of 0.032, and increased in size to 126.6 ± 5.62 nm with a PI of 0.157 following the crosslinking of HA. The charge of the particles also dropped from −5.74 ± 2.24 to −23.64 ± 1.49 mV due to the presence of the excess carboxylic acid groups of the HA on the surface of the liposomes. HA surface decoration was also confirmed by TEM analysis visually depicting the increase in nanoparticle size and surface roughness (Figure 1B). High magnification confocal microscopy was also employed to demonstrate the fluorescent nature of the HALNPs due to the fluorescent tracker in the lipid bilayer (Figure 1C). The rationale for using high molecular weight HA (1.65 MDa) to the LNPs was previous studies have demonstrated that high molecular weight HA is anti-inflammatory and has a high binding affinity to CD44 [19]. Also from our previous study, we demonstrated that crosslinking the LNPs with high MW Hyaluronic Acid (HA) did not increase the particle size and underwent no significant change in net surface charge after lyophilization [22]. We also demonstrated that encapsulation of a range of molecular weight of FITC-tagged Dextran (FD) (10, 20, and 70 kDa) as model drugs did not change the HALNP size. Following nanoparticle design and characterization, we then utilized our HALNP nanocarrier system to examine uptake rate and extent in both GBM and healthy glial cells.

**Table 1:** Size and charge characterization of the lipid nanocarrier pre and post surface decoration with high molecular weight HA

	Hydrodynamic Diameter (nm)	Polydispersity Index	Zeta Potential (mV)
LNP	95.0 ± 0.7	0.032	−5.74 ± 2.24
HALNP	126.6 ± 5.62	0.157	−23.64 ± 1.49

### Preferential uptake of HALNPs by glioblastoma cells over healthy glial cells

The brain is a multi-cell type organ comprising primarily of neurons and a spectrum of glial cells. Consequently, any targeted drug delivery to the brain must facilitate preferential uptake by the cell of interest in order to achieve higher potency, reduce offsite toxicity, and overall promote a positive therapeutic outcome. To test the true merit of HA driven active targeting for GBM therapy, we utilized comprehensive cell cultures consisting of primary rat astrocytes, primary mouse microglia cells, and human GBM cells. In addition to being the most abundant cell type, astrocytes were included in our model system because primary astrocytoma brain tumors including GBM are most similar to astrocyte cells and therefore they represent a targeting hurdle. Furthermore, we included primary astrocytes from both the cortical (Cort Astro) and cerebellum (Cereb Astro) regions of the brain due to their high correlation with GBM onset [58]. Microglial (MG) cells were incorporated in our model system because they are scavenger cells that readily degrade any foreign material in the brain [59], and thus must be evaded for optimal treatment efficacy. Lastly, GBM cells were integrated into our brain model to represent the delivery target. Specifically, three GBM cells lines were selected to obtain results spanning different source donors as well as to probe the potential of treating GBM cells with varying phenotypical properties such as invasive and non-tumorigenic (A172 cells), non-invasive and slightly tumorigenic (U251), and invasive and highly tumorigenic (U87MG) [60].

**Figure 2:**
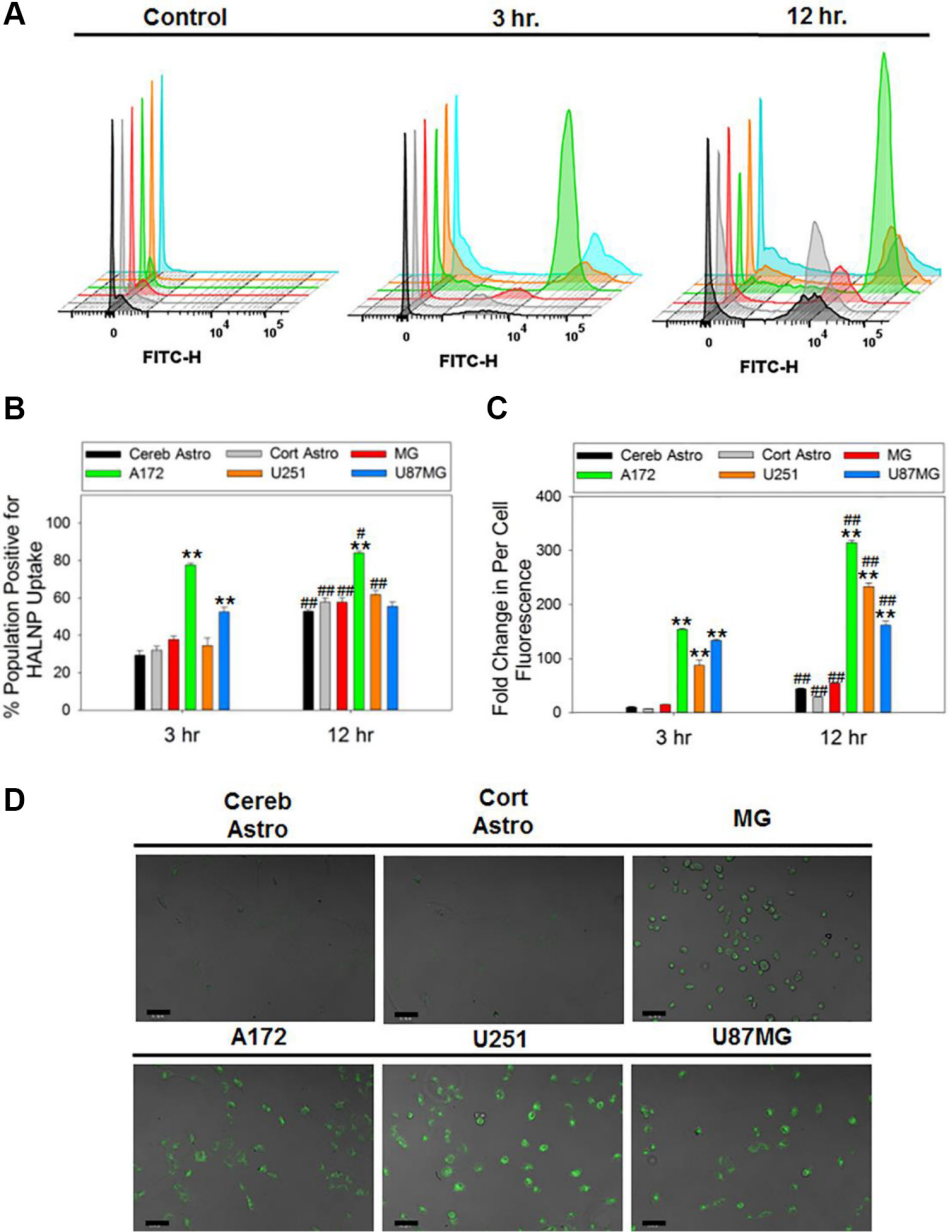
HALNP uptake rate and extent in glioblastoma, astrocytes and microglial cells Flow cytometry was employed to measure HALNP uptake via (**A**) histogram, (**B**) population wide, and (**C**) per-cell fluorescence following a 3 and 12 hour incubation time in cerebellum astrocytes (Cereb Astro), cortical astrocytes (Cort Astro), microglial (MG), and three glioblastoma cell lines (A172, U251, and U87MG) (**p < 0.005, *p < 0.05 relative to both astrocytes and MG at the same time point; ^##^*p* < 0.005, ^#^*p* < 0.05 relative to the same cell type at the previous time point; *n* = 4). For each cell type, control cells (no HALNPs) were used to create a lower limit-gating event to remove cell specific auto-fluorescence. (**D**) Quantitative confocal microscopy was also performed following an analogous three hours incubation time with HALNPs to validate the flow uptake data. The confocal scale bars at 50 micron.

The targeting capacity of HA was directly assessed in GBM cells along with MG and astrocytes using flow cytometry analysis (Figure 2, Supplementary Figure 2). Flow cytometry was chosen due to its ability to examine uptake simultaneously in both a population wide (Figure 2B) and per cell basis (Figure 2C). Prior to analysis, all six cell types were seeded at low density to ensure the uptake data acquired was indicative of per-cell behavior (Supplementary Figure 3). Following a three hour incubation time with the HALNPs, the cerebellum and cortical astrocytes exhibited 29.3% and 32% positive populations for HALNP uptake, and a 10 and 6.7 fold change in per cell fluorescence, respectively. At the same time point, the MG cells had a 37.7% positive population, and a 14.5 fold change in per cell fluorescence, while the A172, U251, and U87MG GBM cells achieved 77.7%, 34.5%, and 52.5% positive populations with 153.4, 87.3, and 133.3 fold change in per cell fluorescence, respectively. This significant difference in HALNP uptake between the glial (astrocytes and microglial) and the GBM cells was further corroborated by quantitative confocal microscopy at the analogous three hour time point (Figure 2D, Supplementary Figure 3).

Flow cytometry was also performed after a 12 hour incubation with HALNPs to reveal if the differential cell uptake patterns were transient or long lived via a preferential mechanism. Following this incubation time, the cerebellum and cortical astrocytes had a 57.8% and 52.9% positive population and a 43.8 and a 28.6 fold change in per cell fluorescence, respectively. Furthermore, the MG cells reached a 57.7% population and a 54.3 fold change in fluorescence, while the A172, U251, and U87MG GBM cells attained 84.1%, 61.8%, and a 55% positive populations with 314.6, 233.3 and 161.7 fold change in per cell fluorescence, respectively. This set of experiments demonstrated the potential of employing HALNPs for targeting GBM cells. In addition, the results obtained seem to indicate that HA may be binding with the GBM cells differently than the healthy glial cells to facilitate preferential intracellular delivery. We have earlier shown that CD44 promotes uptake of HA coated liposomes in breast cancer over corresponding healthy breast tissue [22], and thus we hypothesize this receptor endocytosis route may be a driving force for favored GBM uptake.

### Differential expression of CD44 facilitates active targeting of glioblastoma cells

**Figure 3:**
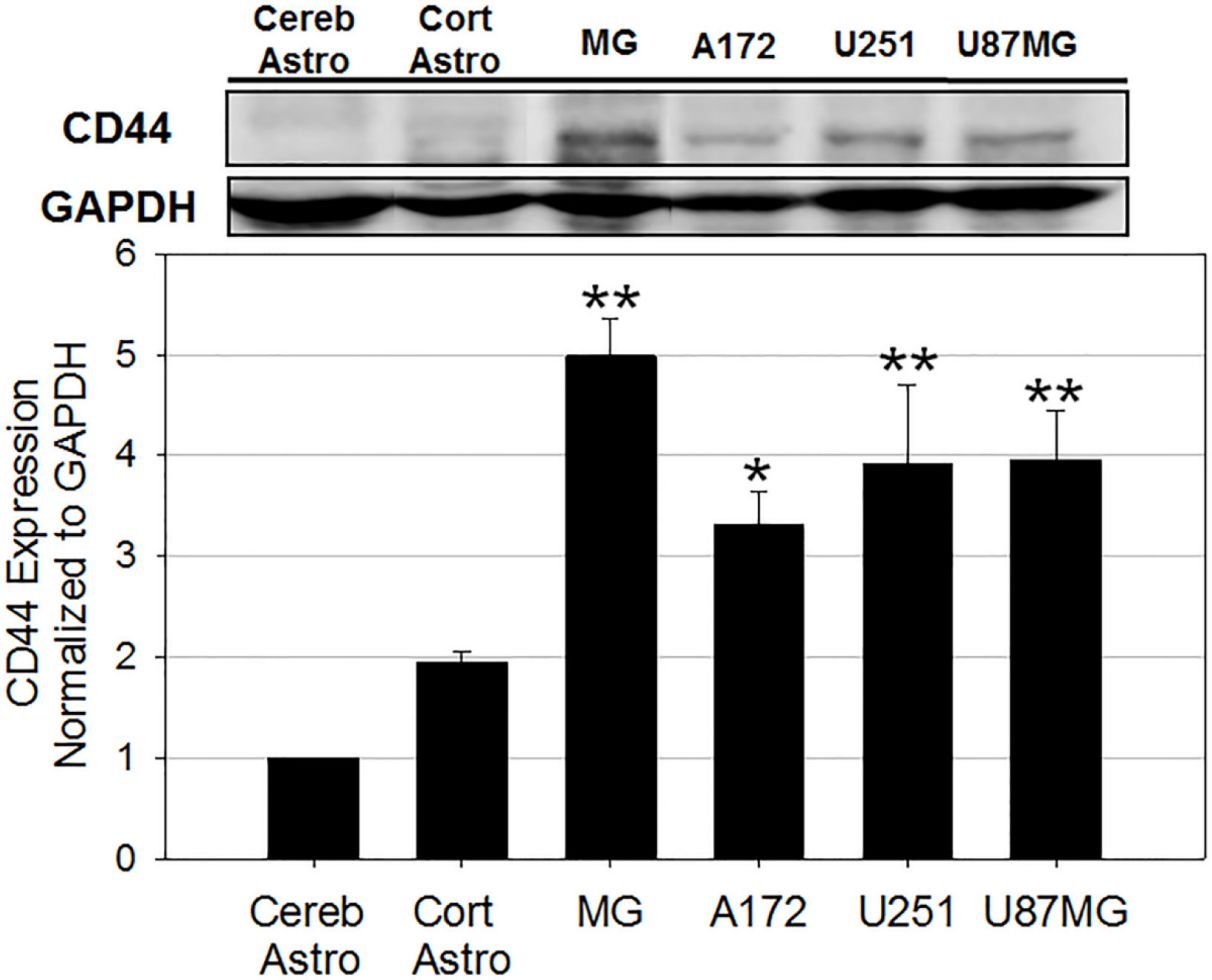
CD44 protein expression analysis in six cells in the GBM tumor microenvironment cerebellum astrocytes (Cereb Astro), cortical astrocytes (Cort Astro), microglial (MG), and three glioblastoma cell lines (A172, U251, and U87MG) (**p < 0.005, *p < 0.05 relative to both Cereb Astro and Cort Astro; *n* = 3). GAPDH was used as the loading control.

CD44 is a cell surface receptor commonly exploited for targeted therapy for a range of cancer types [61]. To investigate if the preferential uptake of HALNPs to GBM cells over astrocytes and MGs is driven by CD44 we first performed western blot to quantify total CD44 protein levels in all six cells types (Figure 3, Supplementary Figure 4). From this analysis we found that CD44 is expressed lowest in both cerebellum and cortical astrocytes, higher in the GBM cells, and highest in the MG cells. By using the cerebellum astrocytes as a comparison baseline for CD44 expression, we found that cortical astrocytes, MG, A172, U251, and U87MG GBM cells had a 2, 5, 3.3, 3.8, and 3.9 fold higher expression, respectively. This data agrees with recent findings that CD44 expression is increased in glioma cells as compared to healthy astrocytes [23, 24]. Furthermore, the high expression of CD44 in MG cells was not surprising because CD44 has been shown to play a role in regulation of macrophage phagocytosis and inflammation pathways [62, 63]. We hypothesize that the CD44 receptors on the MG cells are not functional compared to the ones on GBM cells (at least to high MW HA). Recent studies have demonstrated that CD44 functionality, not necessarily the expression, defines its potential for targeted therapy [64]. We believe that this might be the potential reason for lower uptake of HALNPs in MG cells even when the CD44 expression is the highest in the cells.

**Figure 4:**
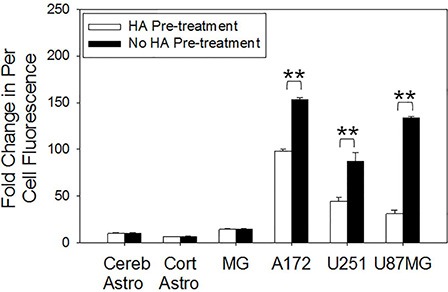
CD44 receptor saturation investigation on the rate of per cell fluorescent HALNP uptake Excess HA was used to saturate CD44 receptors, allowing for the direct quantification of CD44 mediated HALNP endocytosis capacity of each cell type (**p < 0.005, *p < 0.05; *n* = 4). Three hour time point shown.

A saturation experiment was then performed to precisely block the CD44 receptor to discern if the increase in total CD44 expression translated to a higher uptake of the HALNPs (Figure 4). Each of the cell types was pre-treated with excess HA prior to the addition of the HALNPs, and then the per-cell fluorescence was directly compared between the pre-treated and non-pre-treated samples. The percent difference in uptake was then used as a quantitative measure as to the capacity of each specific cell type to employ CD44-HA receptor endocytosis of the HALNPs. From this analysis we found that both cerebellum and cortical astrocytes do not internalize HALNPs via a CD44 driven route, and thus any uptake into these cells must be facilitated by non-specific interactions. Similarly, HA pre-treatment did not significantly influence HALNP uptake in MG cells although they exhibited the highest total CD44 expression. We postulate this is due to the vast amount of different uptake mechanisms employed by MG for immune defense, as well as possible activation state of the MG cells. In contrast, A172, U251, and U87MG GBM cells were found to significantly employ CD44 stimulated uptake yielding a percent difference in per-cell fluorescence of 36%, 48%, and 76%, respectively. Following validation that CD44 facilitates targeted GBM uptake, we next investigated how the CD44 pathway influences intracellular distribution of the HALNPs.

### CD44 stimulated uptake of HALNPs leads to endolysosomal escape in GBM cells

**Figure 5:**
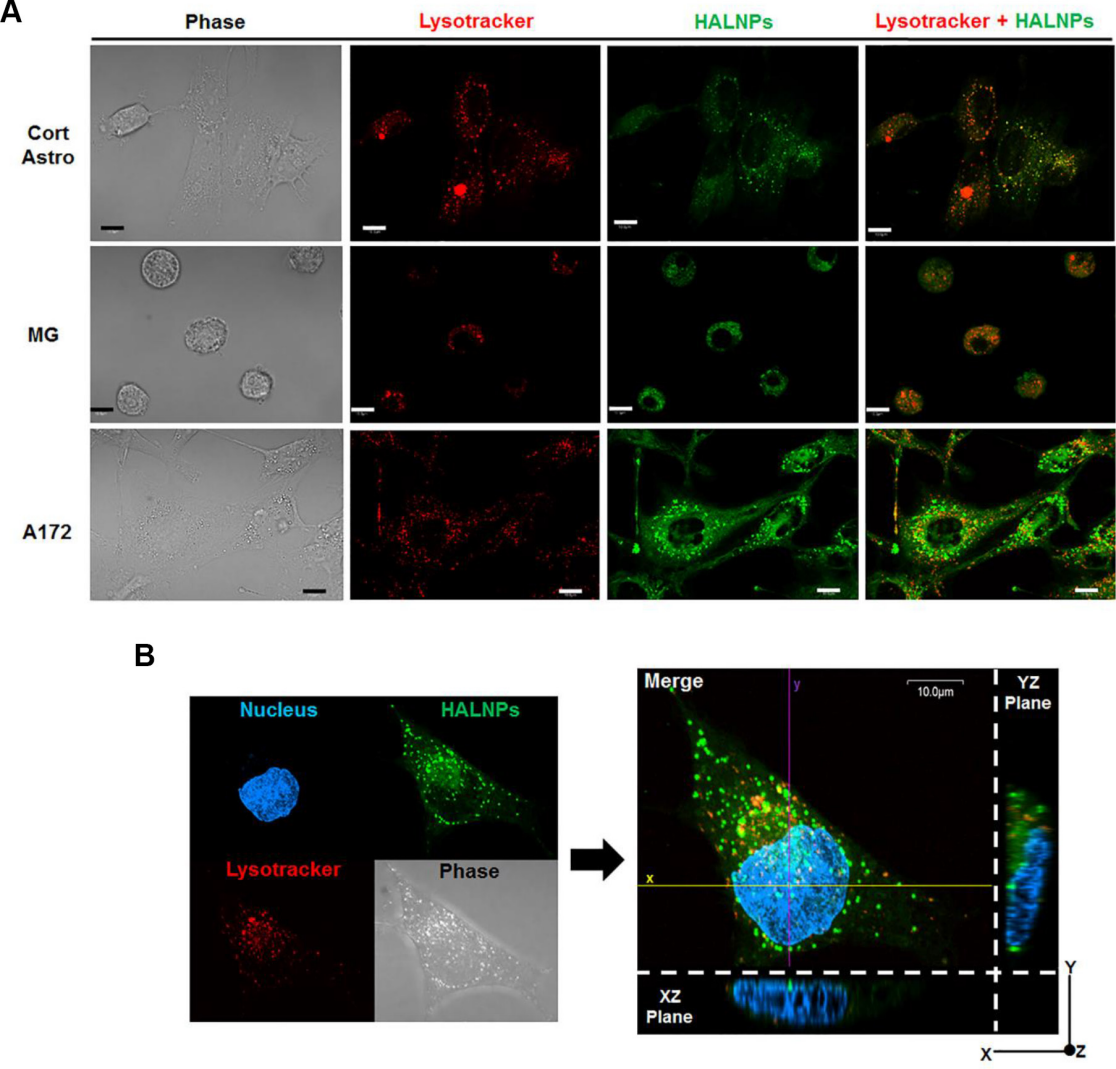
Investigation of HALNP intracellular fate in healthy glial versus GBM cells (**A**) Live cell confocal microscopy with z-axis stacking to probe the HALNP (green) and lysosome (red) co-localization patterns (overlay color = orange). (**B**) Z-axis transformation analysis with optical zoom to validate lysosomal evasion and subsequent achievement of homogenous cytoplasmic distribution in the A172 GBM cell. The cell nucleus was used as an internal reference point (The XZ and YZ planes show the cell height and width, and height and length respectively). For both confocal analyses a five hour incubation time was implemented and the scale bar is 10 micron.

Nanoscale drug delivery systems are commonly hindered in application by inefficient intracellular delivery and subsequent lysosome driven degradation [65]. Therefore, recent efforts have focused on finding ways to simultaneously facilitate targeted uptake and evade lysosome localization [66]. To examine the intracellular distribution of HALNPs as a function of CD44 receptor employment, we performed live cell confocal microscopy to visualize the HALNP-lysosome co-localization patterns. The goal of this experiment was to directly test if cells that exploit CD44 driven uptake (GBM cells) have different HALNP intracellular fate than that of cells that do not readily utilize CD44 (astrocytes, and to a lesser extent MG). For this analysis, we chose cortical astrocytes, MG, and A172 as representative cells for each cell type, and incubated the cells with HALNPs for five hours prior to confocal investigation. Using high magnification confocal microscopy and z axis slicing (Figure 5A), we observed that cortical astrocytes and MG cells displayed extensive HALNP co-localization with lysosomes. This high occurrence of co-localization was expected in cortical astrocytes and MG cells because a majority of non-specific uptake mechanisms except caveolae- culminate their endocytosis pathway by fusion with lysosomes [67]. Alternatively, the A172 cells had significantly less HALNP co-localization with lysosomes following intracellular delivery. This evasion of lysosomal degradation agreed with our previous findings of HALNP intracellular fate with breast cancer cells that also strongly employ CD44 mediated uptake [22], as well as other studies using HA as a targeting moiety [68–70]. To further authenticate that the HALNPs achieved cytoplasmic escape in the A172 cells following CD44 driven uptake, we then performed confocal microscopy with z-axis transformation (Figure 5B). This technique allowed for the direct visualization of HALNPs in comparison to an internal reference frame (in our case the cell nucleus), and fostered confidence that HALNPs achieved homogenous cytoplasmic distribution with minimal lysosomal co-localization. Following this in-depth confocal analysis, we began to hypothesize that the combination of preferential uptake and lysosomal evasion in GBM cells as opposed to the non-specific uptake and lysosomal co-localization of healthy glial cells may lead to enhanced potency and reduced offsite toxicity for HALNP facilitated GBM therapy.

### HALNPs increase the delivery selectivity and overall efficacy of chemotherapeutic GBM therapy

**Figure 6:**
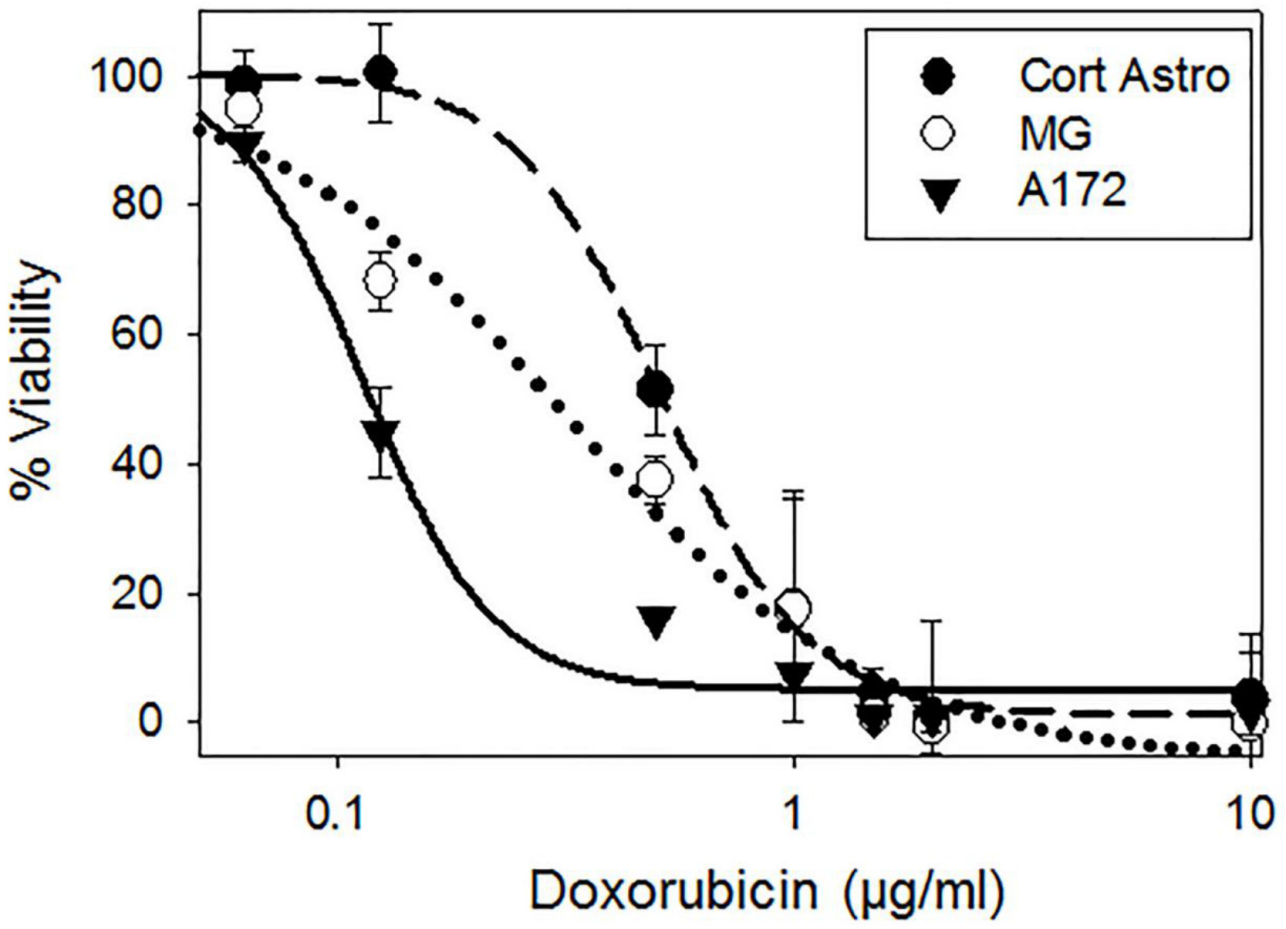
Potency assay employing doxorubicin (DOX) to investigate the influence of HALNP targeting specificity and endolysosomal escape on therapeutic efficacy HALNP encapsulated DOX (HALNP-DOX) potency was compared between glial (cortical astrocytes-Cort Astro and microglial-MG) and GBM (A172) cells following a 24 hour incubation time (*n* = 3).

The therapeutic potential of HA as a targeting ligand for GBM treatment was directly tested by encapsulating Doxorubicin into the HALNPs and performing a 24 hour potency assay with cortical astrocytes, MG, and A172 cells. Doxorubicin (DOX) was chosen as a model chemotherapeutic cargo due to its broad cancer therapy repertoire, including efforts at GBM management [71, 72]. Following our previous protocol for DOX encapsulation into the HALNPs (HALNP-DOX) [57], a Lipid:DOX mass ratio of 2.88:1 was achieved and the final DOX loaded particles were 167.8 ± 9.2 nm in size, −21.19 ± 5.2 mV in charge, and had a PI of 0.241. We seeded the three cell types overnight and then performed a potency assay the next morning over the DOX range (encapsulated DOX) of 0 to 10 μg/ml. Following the 24 hour incubation time, a standard MTT viability assay was used to determine the lethal concentration to kill 50% of the cell population (LC_50_) (Figure 6). This assay demonstrated that the LC_50_ of HALNP encapsulated DOX for the A172 cells was nearly 5 fold lower than the LC_50_ for the cortical astrocytes, and nearly 3 fold lower than the LC_50_ for the MG cells. Specifically, the LC_50_ values were found to be 0.511 ± 0.039, 0.317 ± 0.048, and 0.114 ± 0.010 μg/ml DOX for the Cort astro, MG, and A172 cells, respectively (Table 2). This result was extremely exciting because it clearly shows the significant advantage of employing HA as an active GBM targeting ligand.

**Table 2:** Potency assay with doxorubicin (DOX) LC_50_ values (μg/ml) following the 24 hour incubation time

	Free DOX	HALNP-DOX
Cort Astro	0.322± 0.053	0.511± 0.39
MG	0.267± 0.050	0.317± 0.048
A172	0.193± 0.030	0.114± 0.010

To further probe the specificity of HA mediated targeting, we also performed a 24 hour potency assay with the three cells types utilizing DOX in its free form, not associated with a nanocarrier, over the analogous concentration range (Supplementary Figure 5). From this assay we found that DOX was less potent in the cortical astrocytes and most potent in the A172 cells. The LC_50_ values were measured to be 0.322 ± 0.053, 0.267 ± 0.050, and 0.193 ± 0.030 μg/ml DOX for the Cort astro, MG, and A172 cells, respectively (Table 2). These results are possibly due to the mechanism of action of DOX that includes interfering with the replication process, thus resulting in greater toxicity in more rapidly dividing cells. In addition to providing valuable data regarding the potency of DOX in its free form, this experiment also validated the targeting capacity of HA. By comparing the HALNP-DOX LC_50_ to the free DOX LC_50_ for each specific cell type, the therapeutic influence of specificity was quantified. For the cortical astrocytes, the LC_50_ value significantly increased 59% by utilizing the HALNP system over free DOX. Similarly, LC_50_ value increased by 19% by due to delivery of DOX encapsulated in the HALNPs system to the MG cells. These results exemplify that the HALNP nanoparticles are not readily internalized into healthy glial cells, and thus the HALNP nanocarrier may reduce the chance of offsite toxicity for GBM therapy. However, the LC_50_ value decreased significantly by 41% for the A172 GBM cells via employment of the HALNP system. This experiment confirmed that the HALNP system increases the overall efficacy and safety of chemotherapeutic GBM therapy. Furthermore, the acquired results agree with another study that found that HA targeted liposomes increased the potency of DOX for CD44 positive melanoma, but decreased the potency of DOX when the CD44 active uptake mechanism was removed [73].

## CONCLUSIONS

In conclusion, this study has demonstrated the vast potential of HA facilitated active targeting for GBM nano-therapy. Although the cell surface receptor CD44 has been found to be increased in GBM cells, only a few studies have been conducted implementing HA decorated nanocarriers for GBM treatment. Furthermore, no studies to date have performed an in-depth analysis probing the true merit of HA as a natural ligand to favorably target GBM cells over healthy glial cells. This is the first study known to the authors to employ a comprehensive *in vitro* brain model containing healthy glial cells such as astrocytes and microglia as well as multiple types of glioblastoma cells in combination with a translational HA coated nanocarrier to test the targeting capacity and specificity of HA for GBM treatment. From this analysis we found that HA promotes preferential uptake, facilitates intracellular lysosomal evasion, and significantly enhances chemotherapeutic potency in GBM cells while eluding uptake in astrocytes and MG cells. We believe these findings are significant and will promote the widespread implementation of HA for nanoscale GBM therapy and other brain malignancies. In addition to nanocarriers, we believe these results will also further catalyze CD44 inhibitors as a method for GBM suppression. In the future we plan on harnessing the GBM targeting power of HALNPs with our previously optimized substrate mediated HALNP delivery platform (HALNP-PEM) [57] to create an implantable device to occupy the cavity of resected GBM tumors to promote local, sustained, and targeted therapy and thus bring visible advancement to GBM management.

## MATERIALS AND METHODS

### Materials

For hyaluronic acid coated liposome nanoparticle creation, high molecular weight hyaluronic acid (HA) (~1.65 MDa), 1, 2- Dipalmitoyl-sn-Glycero-3-Phosphoethanolamine (DPPE), Cholesterol (CHOL), and 1-ethyl-3-(3-dimethylaminopropyl) carbomiide (EDAC) were purchased from Sigma Aldrich (St. Louis, MO, USA). Additionally, L α-Phosphatidylcholine (PC), Top Fluor fluorescently conjugated cholesterol (FCHOL), and a mini-extruder apparatus were purchased from Avanti Polar Lipids (Alabaster, AL, USA). For flow cytometry analysis, flow cytometry tubes were purchased from Becton Dickenson (Franklin Lakes, NJ, USA). For cell culture, all tissue culture petri substrates were purchased from Fisher (Waltham, MA, USA).

### Lipid nanoparticle fabrication and hyaluronic acid surface decoration

Multilamellar vesicles were made from PC, DPPE, and CHOL in a 3:1:1 molar ratio and doped with 0.15 mass% FCHOL (as a tracker) by applying the dry lipid film technique, mechanically extruded to the nanoscale, and surface decorated with HA as described earlier [22, 57]. Briefly, extrusion was carried out in a stepwise manner at 65°C ensuring product homogeneity and a final lipid nanoparticle (LNP) diameter in the 80–100 nm range. Following LNP purification via ultracentrifugation to remove any excess lipid debris (1.5 hr, 135,000 g), the primary amine of the DPPE lipid head group was amide bonded to the carboxylic acid group of EDC-activated HA to form HA decorated LNPs (HALNPs) following standard crosslinking protocol. The HALNPs were then purified form excess crosslinking reagents and either 1) stored at 4°C and used within 2 weeks of creation or 2) snap froze and lyophilized following previous protocol for drug entrapment experiments [22]. Lyophilized HA coated particles were observed to be stable for over two months when stored at −80°C (Supplementary Table 1). To determine the effect of HALNP membrane fluidity on the kinetics of drug release, the molar ratio of cholesterol was varied from 20 to 25 % and a model therapeutic cargo, FITC tagged Dextran, was encapsulated into the aqueous core of the HALNPs following previous protocol [22] (Supplementary Figure 6). Although the higher cholesterol formulation extended drug release profiles, this higher cholesterol content was found to reduce encapsulation efficiency. As a result, 20 mol % particles were chosen as the optimized HALNP composition.

### Dynamic light scattering and zeta potential characterization

Hydrodynamic diameter and zeta potential of the LNPs and HA coated LNPs was measured using a Brookhaven NanoBrook ZetaPALS zeta potential and dynamic light scattering instrument (Holtsville, NY, USA). The nanoparticle size was measured as an intensity averaged distribution using a scattering angle of 90°. The Smoluchowki model was utilized to calculate the zeta potential from mobility measurements. All measurements were performed in 0.05 × PBS (pH 7.4) at 25°C.

### Transmission electron microscopy

The phosphotungstic negative stain method was utilized for visualization of the LNP system following previous protocol [22]. A drop of either LNP or HALNP was applied to a carbon film coated copper grid and left to air dry at room temperature. Phosphotungstic acid solution promoted negative staining, and the samples were analyzed in the UNL Microscopy Core Research Facility's TEM model Hitachi H7500 (Chiyoda, Tokyo, Japan).

### Cell culture

All cells were cultured in aseptic conditions following standard cell culture protocol and stored in an incubator set at 37°C with 5% CO_2_.

#### Primary cortical and cerebellum astrocytes

Primary cortical (Cort) and cerebellum (Cereb) astrocytes were prepared from 1–2 day-old Charles River (Wilmington, MA, USA) Sprague-Dawley rat pups from four donor rats yielding 12+ pups per litter in compliance with UNL's IACUC protocol 1046 as described previously [74]. Briefly, the Cort and Cereb brain regions were isolated, digested with trypsin and DNase, filtered through a 70 micron filter, centrifuged, and suspended in complete culture media prior to seeding. The Cort and Cereb Astrocytes were cultured in DMEM/F12 supplemented with 10% FBS and 1% PS from Life Technologies (Grand Island, NY, USA).

#### Primary microglia (MG)

Mouse microglia were grown in macrophage serum free media containing L-glutamine and supplemented with 10% FBS and 1% PS. Additionally, recombinant mouse granulocyte macrophage colony stimulating factor (GM-CSF) from Life Technologies was added to each seeded flask at a concentration of 10 ng/ml and replenished every 3 days.

#### GBM cell lines

A172 (ATCC: CRL1620) human glioblastoma cell line was grown in DMEM/F12 supplemented with 10% FBS and 1% PS. U251 (Sigma) human glioblastoma astrocytoma and U87MG (ATCC: HTB14) human grade IV astrocytoma/glioblastoma cell lines were grown in MEM media supplemented with 10% FBS, 1% PS, 1% NEAA, and 1 mM Sodium Pyruvate (all stated reagents from Thermo Scientific; Waltham, MA, USA).

### Flow cytometry

Flow cytometry was performed employing a FACSCantoII from Becton Dickenson (Franklin Lakes, NJ, USA). Each of the six cell types of the *in vitro* brain model were seeded at a density of 100,000 cells per well in a 12 well plate layout and allowed to incubate overnight to facilitate cell attachment. After this incubation time, HALNPs fluorescently doped with 0.15 mass % FCHOL as a tracker were added to the cells at a concentration of 105 μg lipid per well and incubated for 3 or 12 hours. Directly after this incubation time, the cells were washed three times with sterile 1X PBS, trypsinized, and measured for per cell and population wide fluorescence (ex. 495, em. 520; 10,000 total events/read). By reading control cells without the addition of HALNPs, a lower limit gating event was created to remove cell specific auto fluorescence.

### Confocal microscopy

An Inverted confocal microscope (Olympus IX 81) at the UNL Microscopy Core Research Facility was used for four separate experiments:

#### Confocal of the fluorescently tagged HALNPs

FCHOL doped HALNPs were diluted to 55 ng/ml in 1× PBS and the solution was viewed at 100× magnification with optical zoom using a cover slip (ex. 495, em. 520).

#### Quantitative HALNP Uptake between the *in Vitro* brain model

Cort Astro, Cereb Astro, MG, A172, U251, and U87MG cells were seeded at 230,000 cells per 35 mm glass bottom dish from Mattek (Ashland, MA, USA) overnight to promote cell attachment. The next morning, 1.58 mg/ml HALNP was added per dish and incubated for 3 hours. During the final half hour of the incubation, cellular nuclei were stained with Hoerscht (Thermo Scientific) following stated protocol. Subsequent to the staining procedure, each dish was washed 3× with sterile 1× PBS and kept in HEPES buffered media without phenol red during the confocal visualization. A constant laser intensity was used to take photos at 20× and 60× for each cell type for a quantitative measurement of HALNP uptake.

#### Lysosomal co-localization

Cort Astro, MG, and A172 cells were seeded at 230,000 cells per 35 mm glass bottom dish from Mattek overnight to promote cell attachment. The next morning, 1.58 mg/ml HALNP was added per dish and incubated for 5 hours, and the intracellular lysosomes and nuclei were stained following previous protocol [22]. Following this incubation time, the cells were washed and visualized at 100×. Z-axis slices were merged to display the occurrence of HALNP-lysosome co-localization in each cell type.

#### Z-axis transformation with A172 Cells

A172 cells were seeded at 230,000 cells per glass bottom plate, incubated with 1.58 mg/ml HALNP for five hours, and the lysosomes and nuclei were stained. A Z-axis transformation was then performed (100× with optical zoom) using the stained cell nucleus as an internal reference point. From this analysis, an XZ and YZ plan were constructed to directly visualize cytoplasmic HALNPs.

### Western blot

Total protein was extracted from Cort Astro, Cereb Astro, MG, A172, U251, and U87MG cells cultured on standard TCPS surfaces by RIPA buffer induced cell lysis and protein solubilization followed by the scraping method. Western blotting was used to determine the expression of the cell surface receptor CD44 in each of the six brain cells. BCA protein quantification kit from Abcam (Cambridge, MA, USA) was used to quantify total protein concentration, 25 μg total protein was loaded per lane, the blot was run on a 7.5% tris-glycine SDS PAGE homemade gel, the membrane was probed for CD44 (Abcam; ab24504), and protein bands were developed and quantified by use of a LI-COR (Lincoln, NE, USA) Odyssey FC and Image Studio Lite ver. 5.0 software, respectively. GAPDH (Millipore; ABS16) expression was measured as a normalization control for loading.

### Saturation of CD44 receptor (competition assay)

Cort Astro, Cereb Astro, MG, A172, U251, and U87MG cells were seeded overnight at a density of 100,000 cells per well in a 12 well plate. The next morning, 250 μg of HA was added to select wells and incubated for 1 hour at 37°C. Following this pre-treatment procedure, 105 μg fluorescently doped HALNPs were incubated with the cells for three hours and then flow cytometry analysis was used to directly measure the difference in per cell fluorescence between the HA pre-treated samples and non HA-pretreated samples (of analogous HALNP incubation time and concentration).

### DOX potency assay

#### Encapsulation of DOX into HALNPs

Doxorubicin (DOX) was encapsulated inside the aqueous core of the HALNPs as previously described [57]. Briefly, a vial of lyophilized HALNPs (0.5 mg lipid) was brought to room temperature, and rehydrated with 1/10th its original pre-lyophilized volume of 0.05 × PBS containing 250 μg DOX. Following a thirty minute incubation time to allow for lipid membrane re-assembly, the vial was brought back to its full pre-lyophilized volume with 1 × PBS and ultracentrifuged to remove non-encapsulated DOX (140,000 g, 4°C, and 1.25 hr.). The auto-fluorescent nature of DOX was then employed to determine the entrapment payload. A standard curve consisting of a known amount of DOX was compared to 0.1% triton X-100 permeabilized HALNPs to determine the total encapsulated DOX. A final lipid to DOX mass ratio of 2.88 to 1 was achieved with a 69.4% encapsulation efficiency. These purified DOX containing particles are referred to as HALNP-DOX.

#### Cell seeding and HALNP-DOX addition

Cort Astro, MG, and A172 cells were seeded in a 48 well plate at a density of 35,000 cells per well and incubated overnight to facilitate cell attachment. The next day following validation of homogenous cell attachment, DOX in its free form (not associated with a nanocarrier) or HALNP-DOX were added to select wells in a concentration range of 0 to 10 μg/ml and left to incubate for an additional 24 hours.

#### Free DOX and HALNP-DOX potency determination – MTT assay

To determine the effect of free DOX and HALNP-DOX on the cell viability of Cort Astro, MG and A172 cells, we utilized an MTT assay as previously reported to calculate the lethal concentration to kill 50% of the cells [57, 74, 75]. Following the 24 hour DOX incubation time, the medium of the cells was aspirated, and sterile 5 mg/ml MTT working solution was added and incubated for 2 hours at 37°C. The cells were then lysed with acidified IPA and the absorbance of the produced formazan crystals was measured using a Beckman Coulter AD340 plate reader (Indianapolis, IN, USA). Percent viability was determined by normalization of the 570/620 absorbance ratio to the control untreated cells and positive control dead cells (ethanol treated).

### Statistical analysis

The difference between experimental groups was investigated by a one-way analysis of variance (ANOVA) and by a subsequent Turkey's multiple comparison test in Sigma Plot Software. For statistical analysis of all data, *p* < 0.05 was regarded as the lowest acceptable threshold for significance.

## SUPPLEMENTARY MATERIALS TABLE AND FIGURES


