# Supplementary material for: Hyaluronic acid-conjugated liposome nanoparticles for targeted delivery to CD44 overexpressing glioblastoma cells
Source: Oncotarget. 2016 Apr 22;7(23):34158–71. doi: 10.18632/oncotarget.8926 (PMC5085145; doi:10.18632/oncotarget.8926)
Supplement: Supplementary file 1 [file oncotarget-07-34158-s001.pdf]

Hyaluronic acid-conjugated liposome nanoparticles for targeted delivery to CD44 overexpressing glioblastoma cells

Supplementary Materials

Supplementary Table S1: HALNPs temporal stability analysis in the lyophilized state following standard rehydration protocol

	Hydrodynamic Diameter (nm)	Polydispersity Index (PI)
Day 0	167.2 ± 2.3	0.185
Day 30	169.2 ± 4.3	0.160
Day 60	171.4 ± 6.6	0.172

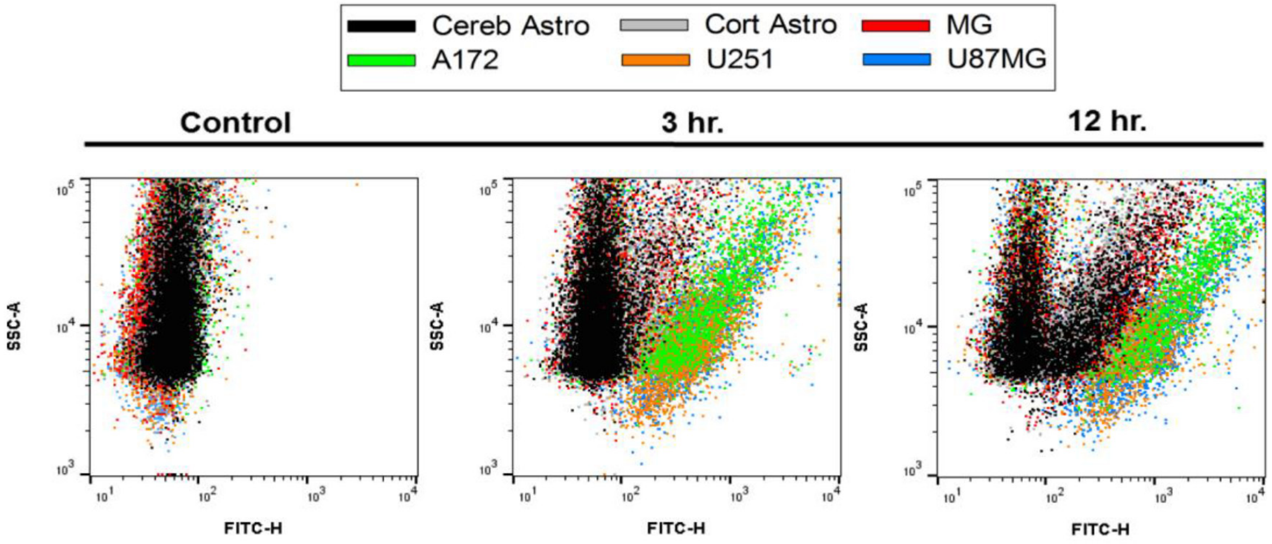

Supplementary Figure S1: HALNP uptake rate and extent in glioblastoma and healthy glial cells. Flow cytometry scatter plot analysis was used to display HALNP uptake following a 3 and 12 hour incubation time with the *in vitro* GBM model.

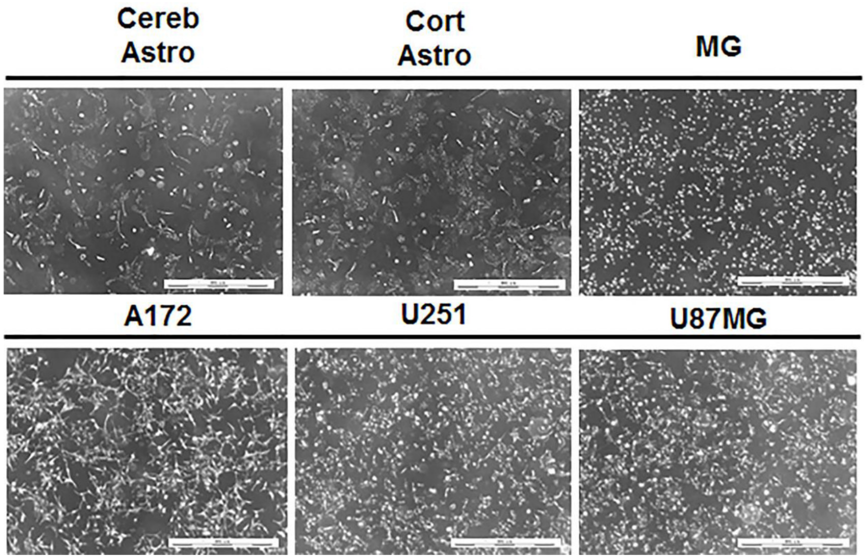

Supplementary Figure S2: Seeding density for flow cytometry experiments. Scale bar is 1000 micron.

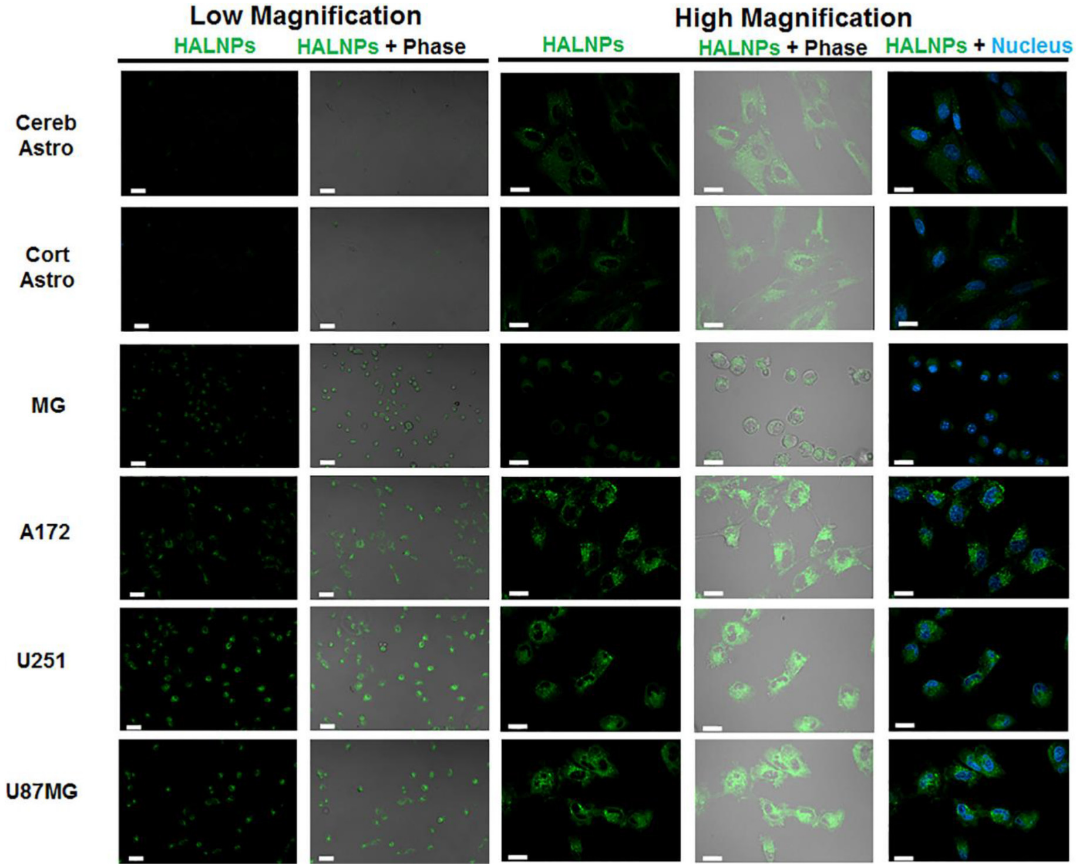

Supplementary Figure S3: Expanded live cell quantitative confocal microscopy analysis of HALNP uptake between healthy glial (Cereb Astro, Cort Astro, and MG) and GBM (A172, U251, U87MG) cells following a five hour incubation time. Low magnification scale bar is 50 micron. High magnification scale bar is 20 micron.

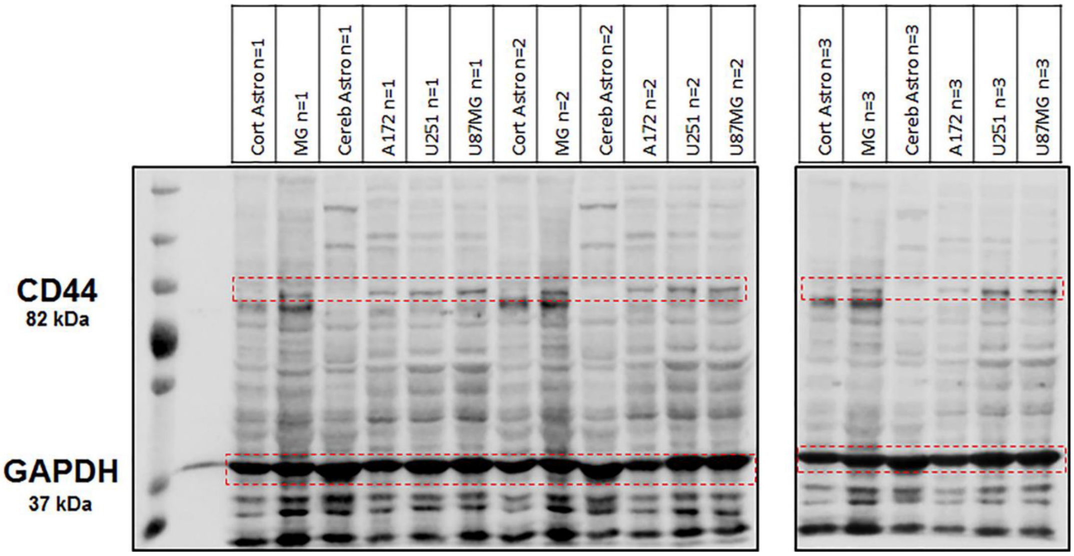

Supplementary Figure S4: CD44 protein analysis full western blots un-cropped. CD44 and GAPDH proteins are labeled. The red boxes indicate the protein bands used for quantification; *n* = 3.

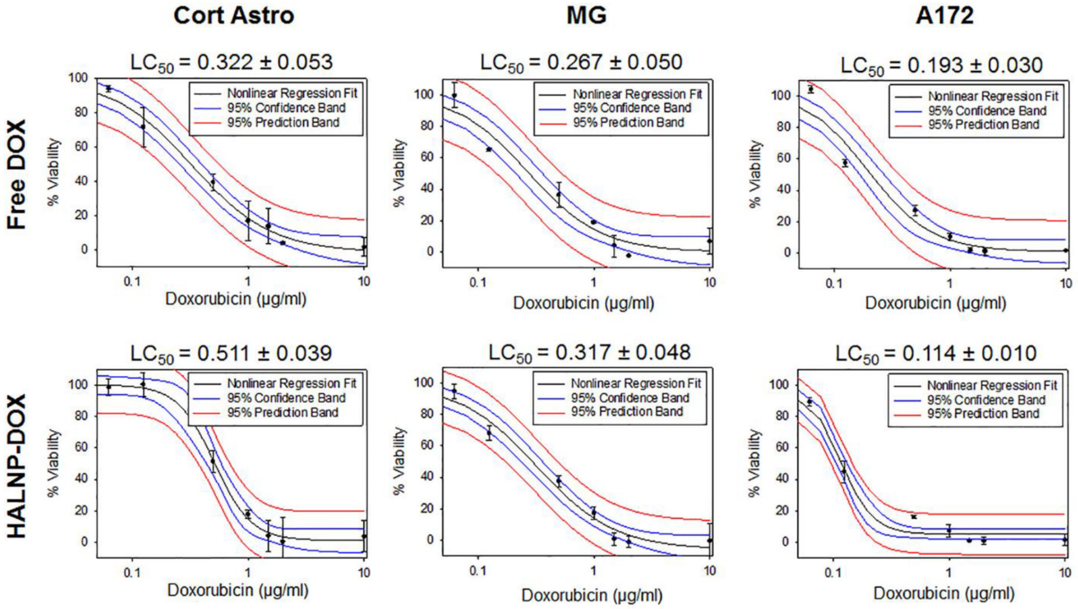

Supplementary Figure S5: Potency assay LC_{50} nonlinear regression analysis with both confidence and prediction bands for the 24 hour time point employing Doxorubicin in its free form (Free DOX) and encapsulated inside HALNPs (HALNP-DOX).

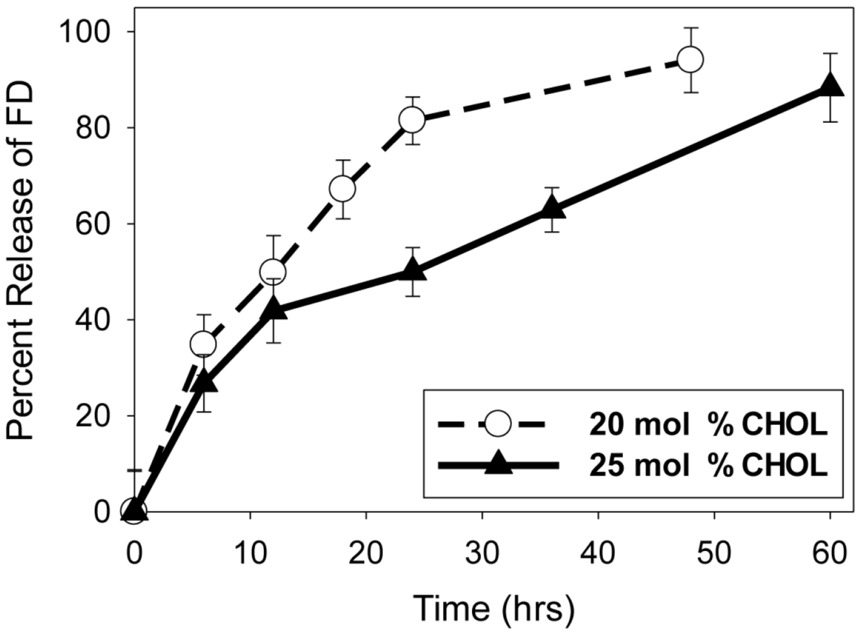

Supplementary Figure S6: Effect of HALNP cholesterol content on FITC-Tagged Dextran (FD) model drug cargo release.
